# Balanced Crystalloids versus Normal Saline in Children with Critical Asthma

**DOI:** 10.3390/children9101480

**Published:** 2022-09-28

**Authors:** Andrea Scioscia, Christopher Horvat, Michael L. Moritz, Dana Fuhrman

**Affiliations:** 1 Department of Pediatrics, UH Rainbow Babies and Children’s Hospital, Cleveland, OH 44106, USA; 2 Department of Critical Care Medicine, UPMC Children’s Hospital of Pittsburgh, Pittsburgh, PA 15224, USA; 3 Department of Pediatrics, UPMC Children’s Hospital of Pittsburgh, Pittsburgh, PA 15224, USA

**Keywords:** balanced crystalloids, normal saline, sodium chloride, pediatric, asthma, maintenance fluids

## Abstract

There is little known about the impact of maintenance fluid choice in children with critical asthma on clinical outcomes. Our primary study objectives were to determine the differences in the serum chloride and bicarbonate levels based on the receipt of 0.9% saline or a balanced solution. The secondary study objectives included differences in acute kidney injury (AKI) and intensive care unit (ICU)/hospital length of stay (LOS). In this retrospective cohort study, we included 1166 patients admitted to a quaternary children’s hospital with critical asthma between 2017 and 2019. The patients were stratified based on if they received 0.9% saline or a balanced solution (Lactated Ringer’s or Plasma-lyte) for maintenance therapy. The study outcomes were determined using independent sample *t*-tests, multivariable logistic regression, and negative binomial regression. The patients who received 0.9% saline maintenance therapy had a significantly higher increase in their serum chloride levels when compared to those who received balanced solutions (0.9% saline: +4 mMol/L, balanced: +2 mMol/L, *p* = 0.002). There was no difference in the decrease in the serum bicarbonate levels (0.9% saline: −0.4 mMol/L, balanced: −0.5 mMol/L, *p* = 0.830). After controlling for age, race, sex, and the Pediatric Logistic Organ Dysfunction (PELOD-2) score, there was no association between the type of fluid received and the development of AKI (OR 0.87, 95% CI: 0.46–1.63, *p* = 0.678). Additionally, there was no association between the type of fluid and hospital or ICU LOS. Thus, despite higher serum chloride levels in the patients that received 0.9% saline, the choice of fluid therapy did not have an impact on the serum bicarbonate values, the development of AKI or hospital and ICU LOS, suggesting there is little difference between 0.9% saline and balanced solutions as maintenance therapy in children with critical asthma.

## 1. Introduction

The association of hypotonic (0.45%) saline with hyponatremia in hospitalized children is currently well established in the pediatric literature [1,2,3,4], such that the 2018 clinical practice guidelines [5] recommend the use of isotonic saline (0.9%) as the fluid of choice for maintenance therapy in pediatrics. There is also a body of evidence suggesting that balanced solutions rather than 0.9% saline as a resuscitative fluid may have the potential to reduce morbidity and mortality for critically ill patients [6,7,8]. Zampieri et al., however, have recently shown that this may not be the case and that, in fact, the two may have near equal efficacy [9].

No prior study’s results have demonstrated the sole impact of isotonic saline when compared to balanced solutions as maintenance therapy, following initial resuscitation. In the pediatric intensive care unit (ICU), we see a variety of patients with underlying comorbidities that disrupt their electrolyte and/or acid-base homeostasis. Many of these patients require fluid resuscitation prior to arrival to the ICU, thus complicating the ability to isolate the effect of maintenance intravenous (IV) fluids on electrolyte disturbances. Patients admitted with critical asthma, however, often do not have multiple comorbidities and do not require high volumes of fluid resuscitation. They do require close monitoring of their intake and output, as patients with critical asthma often present dehydrated with increased insensible losses. Administering too much fluid, however, can increase their risk for transpulmonary edema [10]. Thus, their lack of comorbidities and their strict monitoring of intake and output make them a favorable group to investigate the effects of isotonic and balanced fluids as maintenance therapy.

Our primary study objective was to evaluate the incidence of hyperchloremia and low bicarbonate levels for children with critical asthma who had received maintenance fluid therapy with a balanced solution (Lactated Ringer’s (LR) or Plasma-lyte) versus 0.9% saline. For secondary outcomes, we looked at the development of AKI, ICU and hospital LOS. We hypothesized that the incidence of hyperchloremic metabolic acidosis (HCMA) would be greater in patients given 0.9% saline as compared to those given balanced solutions as maintenance therapy. We anticipated that the use of 0.9% saline would be associated with AKI and a longer hospital and ICU length of stay.

## 2. Materials and Methods

In this retrospective cohort study, we included 1166 patients admitted with critical asthma to the pediatric ICU at our quaternary children’s hospital between 2017 and 2019. The study was approved by the Institutional Review Board for Health Sciences Research at the University of Pittsburgh. By the definition of asthma, patients under two years of age were excluded [11]. We also excluded patients under 20 kg, so as to standardize the volume of maintenance fluid administered for purposes of the analysis. Patient demographics were extracted from the UPMC Children’s Hospital of Pittsburgh’s electronic health records, and patients were selected based on their ICD-10 diagnosis codes. All laboratory tests were performed in the clinical laboratory. The Pediatric Logistic Organ Dysfunction (PELOD-2) score was determined for each patient at the time of admission [12]. 

We stratified patients by the type of maintenance fluid received at the time of admission: 0.9% saline versus a balanced solution, either LR or Plasma-lyte. The fluid administered was at the discretion of the primary PICU physician. Baseline characteristics were analyzed, such that categorical variables were summarized as number and percent and were compared using a Chi-square analysis. Non-normally distributed continuous variables were reported as median and interquartile range and compared using Wilcoxon rank sum, while normally distributed continuous variables were reported as mean with standard deviation (SD) and compared using independent samples *t*-tests.

We examined the development of acute kidney injury (AKI), using the KDIGO serum creatinine criteria [13]. Admission creatinine was used as baseline creatinine. To evaluate the association of maintenance therapy fluid choice on AKI, we used multivariable logistic regression. The association of maintenance therapy fluid choice and ICU and hospital length of stay was examined with negative binomial regression. The significance level was set at <0.05. All data were analyzed with R (R Foundation, Vienna, Austria) version 4.1.1 and RStudio version 1.4 (RStudio, Boston, MA, USA). 

## 3. Results

Of the 1166 patients included in our study, there were no differences in the baseline characteristics between the 0.9% saline and balanced solution groups (Table 1).

### 3.1. Primary Outcomes

**Figure 1:**
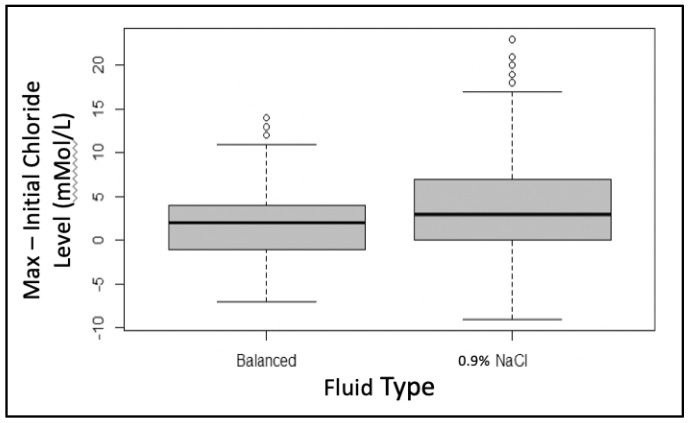
Comparison of the change in serum chloride level by subtracting the initial chloride level from the maximum chloride level in the first 72 h of admission. The 0.9% saline group had a significantly higher increase in chloride level compared to the balanced solution group, *p* < 0.005.

**Figure 2:**
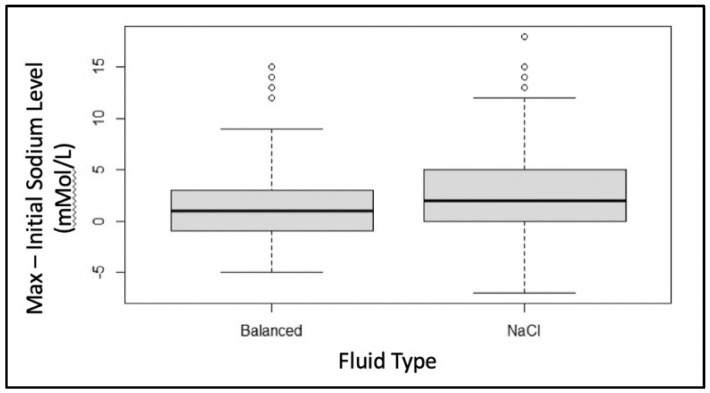
Comparison of the change in serum sodium level by subtracting initial sodium level from the maximum sodium level in the first 72 h of admission. The 0.9% saline group had a significantly higher increase in sodium level compared to the balanced solution group, *p* < 0.005.

The starting mean (SD) serum chloride level between the two groups differed slightly (0.9% saline = 108 (5) mMol/L vs. balanced = 109 (4) mMol/L, *p* = 0.031). After subtracting each individual’s admission chloride level from their maximum chloride level in the first 72 h of admission, we saw that those who had received 0.9% saline maintenance therapy had a significantly higher increase in their chloride levels when compared to those who received balanced solutions (+4 mMol/L vs. +2 mMol/L, *p* < 0.005) (Figure 1). The change in the serum sodium was compared in a similar manner, and we noted that the 0.9% saline group also had a significantly higher increase in their sodium levels (*p* < 0.005) (Figure 2). 

**Figure 3:**
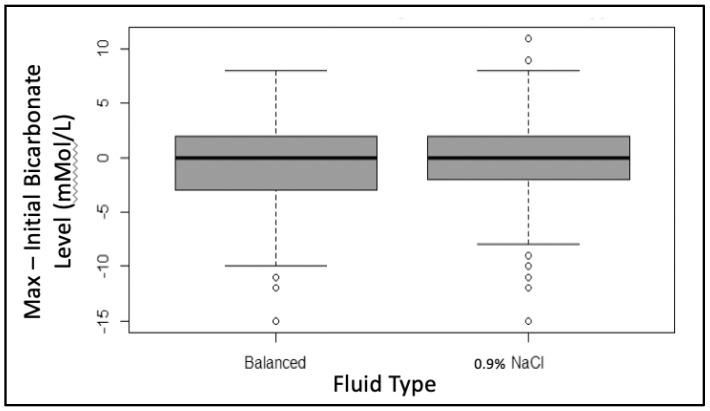
Comparison of the change in serum bicarbonate level by subtracting the minimum bicarbonate level from the initial bicarbonate level in the first 72 h of admission. There was no difference in the decrease in bicarbonate levels between the two groups, *p* = 0.830.

The same analysis was performed to evaluate for a change in the serum bicarbonate level. The starting bicarbonate level did not differ between the two groups (0.9% saline: 19 (6) mMol/L vs. balanced 20 (4) mMol/L, *p* = 0.294). When compared, there was no difference in the decrease in the bicarbonate levels between the two groups (−0.4 mMol/L vs. −0.5 mMol/L *p* = 0.830) (Figure 3). 

### 3.2. Secondary Outcomes

There were 531 patients who had more than one creatinine level recorded. Of those, 63 patients met the criteria for at least stage 1 AKI. After controlling for age, sex, race, and admission PELOD-2 score (Table 2), we found no association between AKI and the maintenance fluid therapy administered (OR 0.87 (95% CI: 0.46–1.63), *p* = 0.678) (Table 2). Further, only nine patients in the 0.9% saline group and five patients in the balanced group developed either stage 2 or 3 AKI. 

With a negative binomial regression model, we again controlled for age, race, sex, and admission PELOD-2 score. Once more, there was no association between the type of fluid and hospital LOS (Median (IQR)—0.9% saline: 1.3 days (1.78) vs. balanced: 1.5 days (2.1), *p* = 0.492) or ICU LOS (Median (IQR)—0.9% saline: 2.9 days (3.1) vs. balanced: 3.2 days (3.8), *p* = 0.138).

## 4. Discussion

We found that there was a significant increase in both the serum chloride and sodium levels for the 0.9% saline group when compared to the balanced solution group without a greater decrease in the bicarbonate levels. Consistent with this finding, we did not see a difference in the rate of AKI when comparing the two groups. Our findings suggest that despite a greater rise in chloride levels from receiving a maintenance fluid containing a greater chloride content, there is no significant effect on clinically relevant outcomes, such as AKI or LOS. 

The starting chloride values for each group (0.9% saline = 108 mMol/L and balanced = 109 mMol/L) could be characterized as hyperchloremic, even before the initiation of maintenance therapy. For this reason, we could not characterize the “development” of hyperchloremia, rather we assessed the change in chloride level. The reason for elevated chloride levels at the initiation of therapy is not entirely clear. We did not account for any resuscitation fluid prior to arrival. If patients received bolus fluids with 0.9% saline ahead of time, this certainly could have played a role, again complicating any interpretation regarding the “development” of hyperchloremia. Further, the tendency toward more severe hyperchloremia for the normal saline group was reflective of an increase in serum sodium rather than the development of metabolic acidosis.

Even if there is a tendency toward more severe hyperchloremia for the 0.9% saline group, this may not be indicative of its impact on the development of a metabolic acidosis in this population in the first 72 h of PICU admission. This subset of patients, like many critically ill patients, tends to produce increased levels of vasopressin during their acute illness [1,14]. This can lead to free water retention and hyponatremia. For this reason, we examined the change in the sodium levels as well and found there was actually a similar increase (Figure 2). Therefore, this is likely indicative of the fluid received. If they had decreased, however, we would have anticipated another reason for increased losses, such as AKI. We did not find any association between the maintenance fluid therapy received and the development of AKI. Because we also did not see any impact on the development of hyperchloremic metabolic acidosis, this is probably not surprising. 

These findings have potentially important implications for clinical practices. Normal saline fluids can contribute to iatrogenic hyperchloremia and a resulting non-gap metabolic acidosis, theoretically exacerbating tachypnea and potentially worsening the work of breathing in the setting of existing respiratory distress. In contrast, balanced fluids may mitigate this risk of hyperchloremia while avoiding iatrogenic hyponatremia. Conversely, balanced fluids are more often incompatible with medications commonly administered in the setting of respiratory distress, such as ceftriaxone for the treatment of community-acquired pneumonia. The use of balanced fluids may therefore contribute to the placement of additional vascular access and add to a child’s discomfort during hospitalization. While this study did not focus on measurements of respiratory rate or objective markers of respiratory distress among children who received 0.9% saline versus balanced fluids, there was no significant difference in the patient-centered outcomes of adjusted ICU or hospital LOS, suggesting that iatrogenic hyperchloremia does not substantially prolong symptoms of distress in children with respiratory disease. 

Our study was limited in that it was a retrospective study completed at a single center. Additionally, we did not stratify patients based on any fluid received in the emergency department or prior to arrival to our unit. We also did not include this resuscitation fluid in our analysis. We acknowledge that there may be additional differences in pre-treatment characteristics between the two groups, including underlying co-morbidities, which were unaccounted for in our analysis. While we included the PELOD-2 scores as a measure for illness severity, this may not have been the most appropriate measure for our population, as most of these children would not suffer end-organ damage. Instead, it may be more appropriate to look at asthma severity scores or starting therapy doses (i.e., starting albuterol dose) as a better marker for illness severity. Our study was limited to patients with critical asthma. It is possible that our findings are not applicable to other patient groups receiving maintenance fluid therapy. 

## 5. Conclusions

We found that the increase in chloride values in children who received 0.9% saline for maintenance therapy with critical asthma was greater when compared to those that received balanced solutions. This was not, however, associated with a decline in bicarbonate levels. We found no evidence that the use of balanced solutions resulted in improved outcomes, such as AKI, ICU, or hospital LOS, for children with critical asthma. 

## Figures and Tables

**Table 1:** Baseline characteristics at time of PICU admission. Categorical variables are summarized as number (%) and compared with Chi-square analysis. Normally distributed variables are reported as mean (SD) and compared with independent samples *t*-test, while non-normally distributed continuous variables are reported as median (IQR) and compared with Wilcoxon rank sum. Refer to Appendix A for the Asthma Distress Score criteria.

Baseline Characteristics at Time of PICU Admission			
	0.9% Saline (n = 751)	Balanced (n = 415)	*p*-Value
Sex Male Female	424 (56%) 327 (44%)	228 (55%) 187 (45%)	
Race Caucasian Non-Caucasian	455 (61%) 296 (39%)	253 (61%) 162 (39%)	
Age (Years)	11.3 (4.9) ^^^	11.1 (4.8) ^^^	*p* = 0.502
PELOD-2 Score	4.0 (2.0–6.0) ^&^	4.0 [2.0–6.0] ^&^	*p* = 0.029
Asthma Distress Score *	6.4 (3.2) ^^^	6.7 (3.2) ^^^	*p* = 0.324
Chloride Level (mMol/L)	108 (5) ^^^	109 (4) ^^^	*p* = 0.031
Bicarbonate Level (mMol/L)	19 (6) ^^^	20 (4) ^^^	*p* = 0.294

^ Mean (SD), & Median [25–75%], * Appendix A.

**Table 2:** Multivariable Logistic Regression Analysis for the Development of Acute Kidney Injury (AKI). There was no association between maintenance fluid therapy choice and AKI. (OR: odds, ratio, PELOD-2: Pediatric Logistic Organ Dysfunction).

Characteristic	OR (95% CI); *p*-Value
Maintenance Fluid Therapy	0.87 (0.45–1.63); *0.678*
Sex	0.80 (0.42–1.51); *0.501*
Race	0.56 (0.30–1.04); *0.064*
Age (Years)	0.98 (0.92–1.04); *0.499*
Admission PELOD-2 Score	1.23 (1.21–1.37); *<0.001*

**Figure A1:**
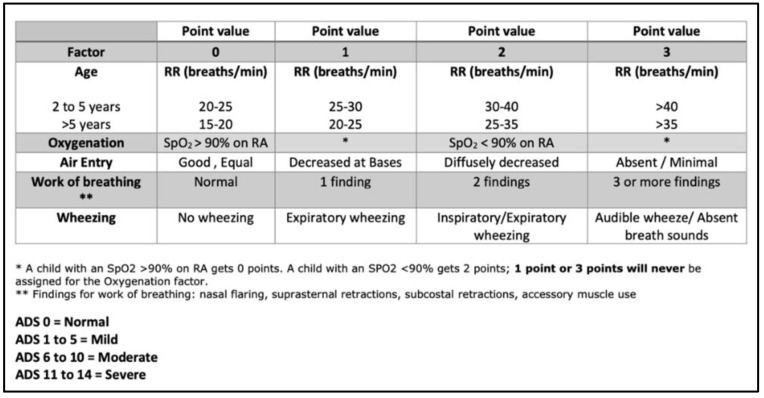
Asthma Distress Score. This is the criteria utilized by the respiratory therapy department at the UPMC Children’s Hospital of Pittsburgh [15].

## Data Availability

Due to privacy restrictions, data may be available upon request.
